# Supplementary figures and images for: Low-dose mixtures of dietary nutrients ameliorate behavioral deficits in multiple mouse models of autism
Source: PLoS Biol. 2025 Dec 2;23(12):e3003231. doi: 10.1371/journal.pbio.3003231 (PMC12671748; doi:10.1371/journal.pbio.3003231)

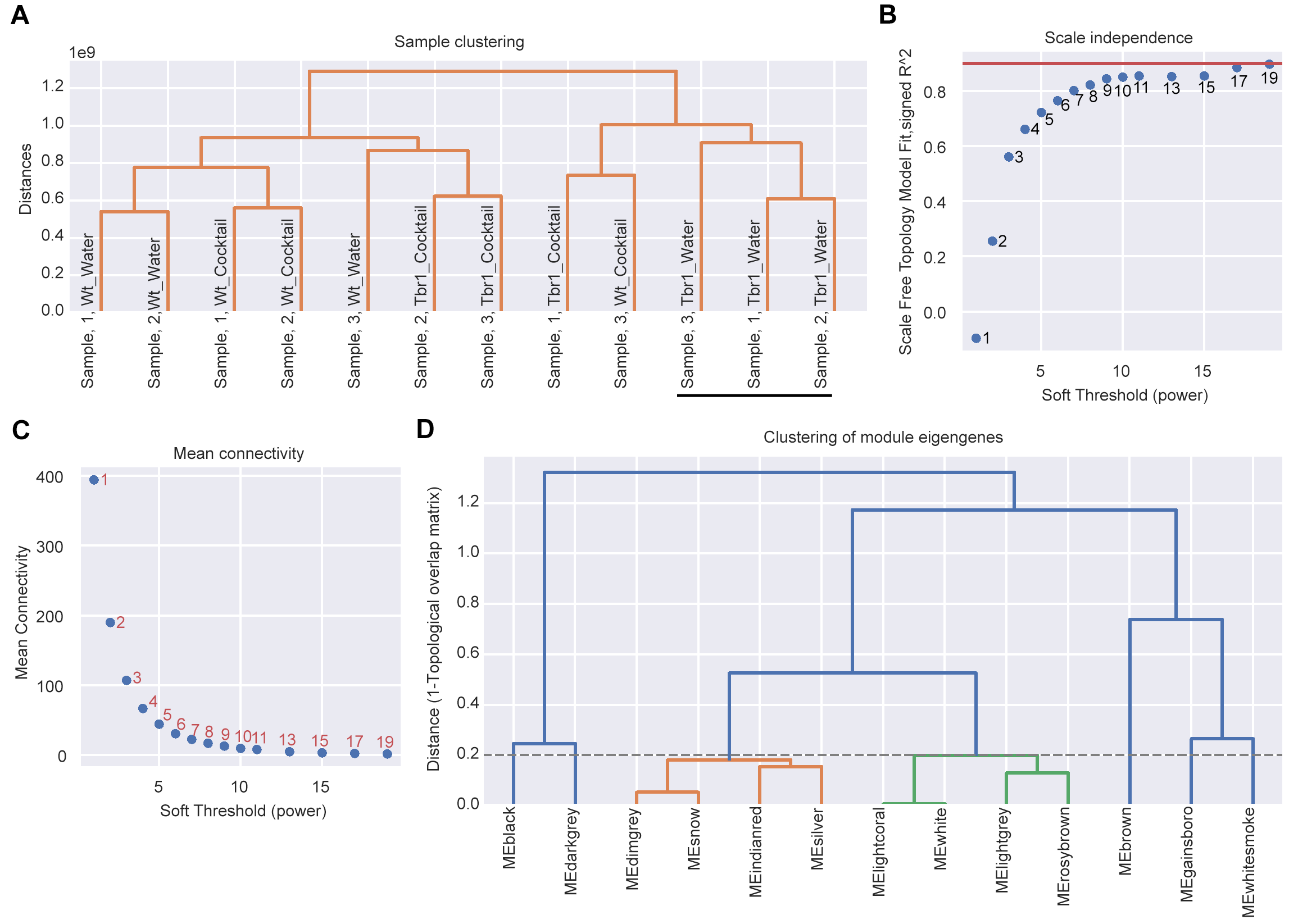

Supplement: S1 Fig — (A) Sample clustering. The three samples of the Tbr1_Water group were closer to each other and distinct from the other samples. (B–C) Checks of the soft-thresholding power for network topology analysis. (D) Clustering of module eigengenes. The cutoff value of distance was set at 0.2. Thus, modules with a distance <0.2 were merged into one. Related to Fig 1. The entire list of the selected proteins for PyWGCNA can be found in S1 Data. (TIF) [file pbio.3003231.s001.tif]

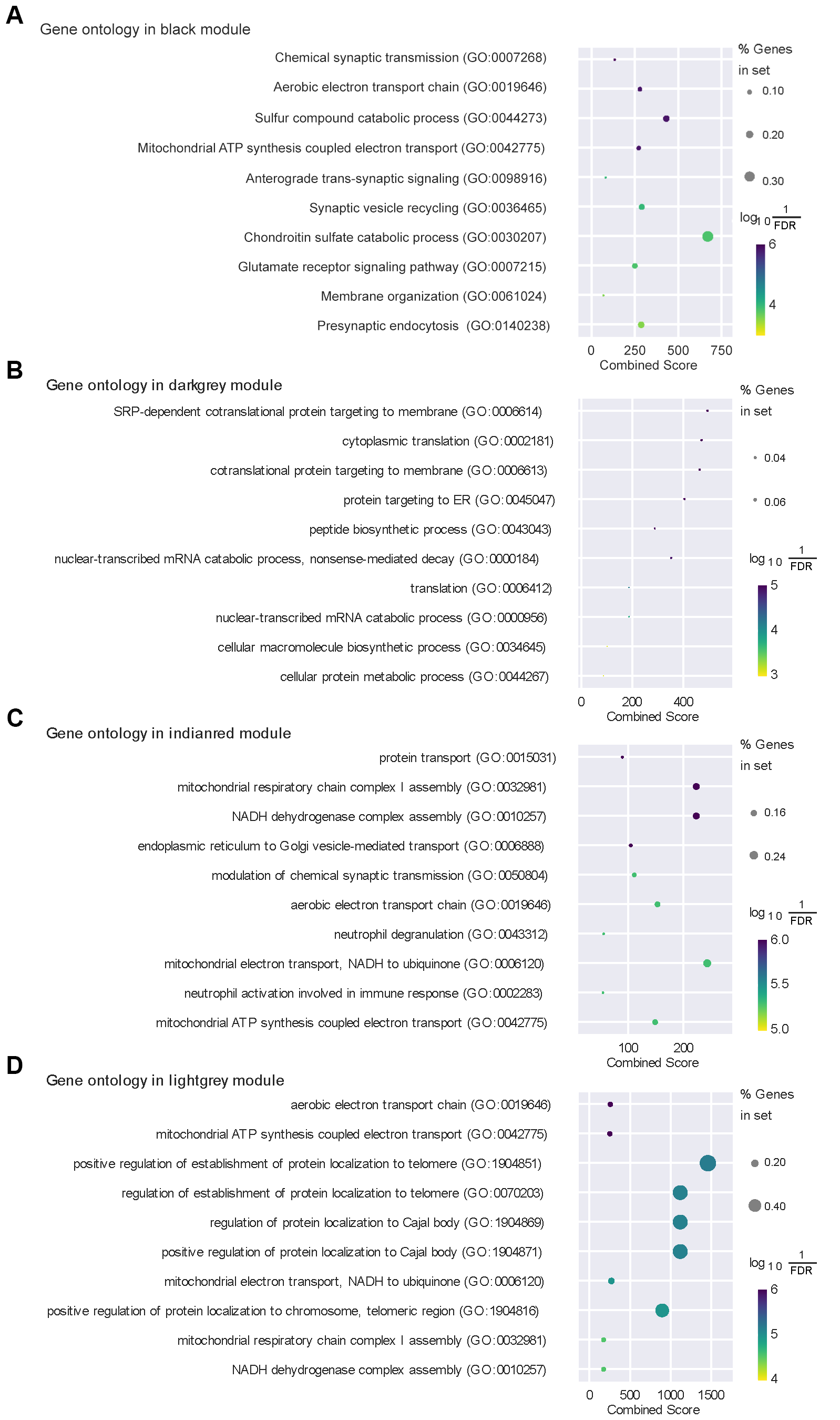

Supplement: S2 Fig — (A) Black, (B) Dark gray, (C) Indian red, and (D) Light gray eigengenes. Related to Fig 1. All selected proteins of Black module are available in S2 Data. (TIF) [file pbio.3003231.s002.tif]

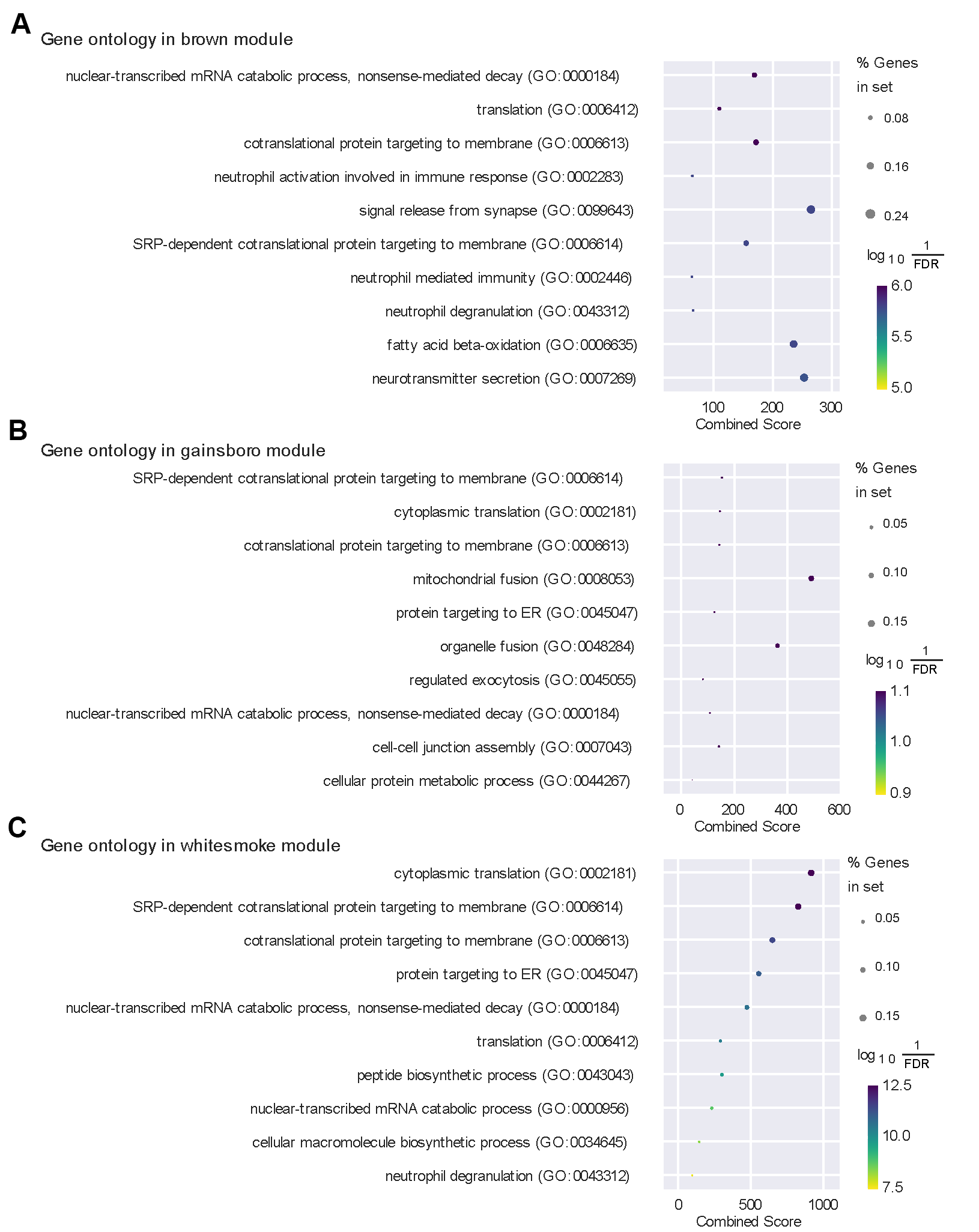

Supplement: S3 Fig — (A) Brown, (B) Gainsboro, and (C) Whitesmoke eigengenes. Related to Fig 1. (TIF) [file pbio.3003231.s003.tif]

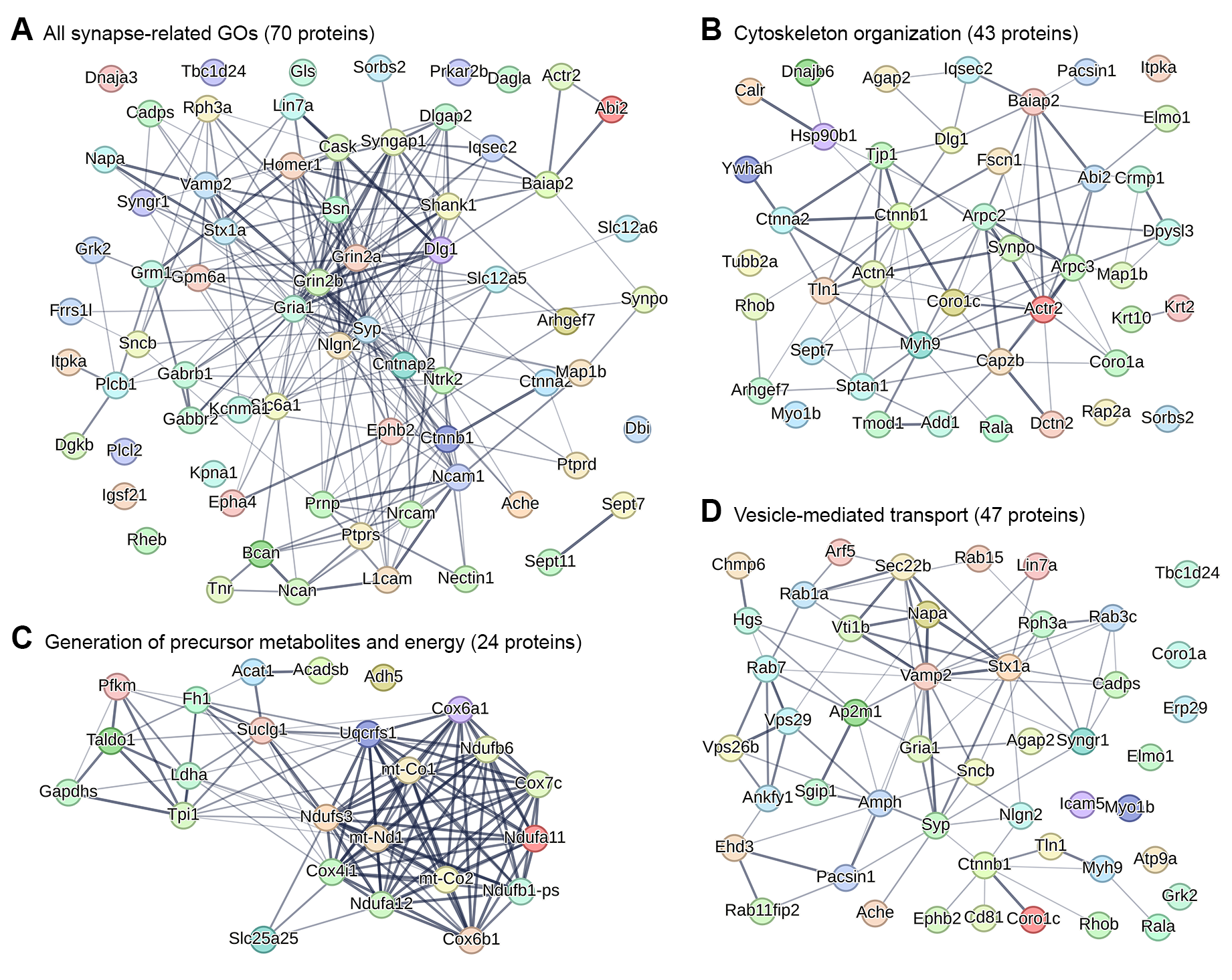

Supplement: S4 Fig — (A) All synapse-related GO (70 proteins). (B) Cytoskeleton organization-related GO (43 proteins). (C) Proteins related to the GO term of generation of precursor metabolites and energy (24 proteins). (D) Vesicle-mediated transport GO (47 proteins). Related to Fig 1. (TIF) [file pbio.3003231.s004.tif]

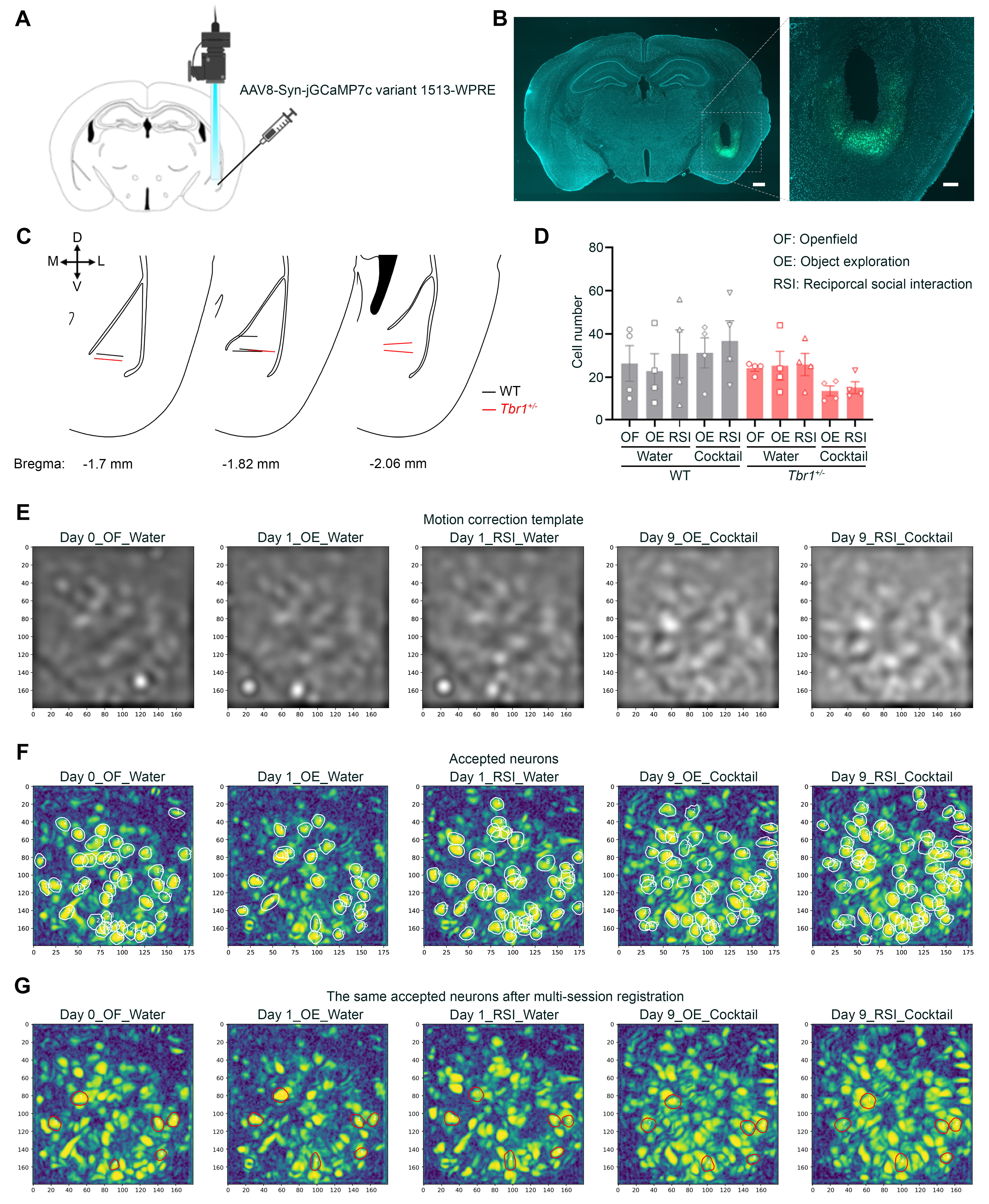

Supplement: S5 Fig — (A) Schematic of virus injection and GRIN lens implantation. (B) An example of a mouse brain infected with AAV and implanted with a GRIN lens. (C) Positioning of the GRIN lens in WT and Tbr1+/− mice (WT: n = 4; Tbr1+/−: n = 4). D, dorsal; V, ventral; M, medial; L, lateral. (D) Number of firing neurons in WT and Tbr1+/− mice across sessions. Data is represented as mean ± SEM. Each dot represents an individual mouse. (E–G) Processing of calcium images using CaImAn. (E) Template for motion correction. (F) Accepted firing neurons are labeled by white circles. (G) The same accepted firing neurons after multi-session registration are labeled by red circles. Related to Fig 4. The data underlying the graph of (D) can be found in S3 Data. The statistical results are available in S4 Data. The figure was created in BioRender. Lin, M. (2025) https://BioRender.com/akrum83. (TIF) [file pbio.3003231.s005.tif]

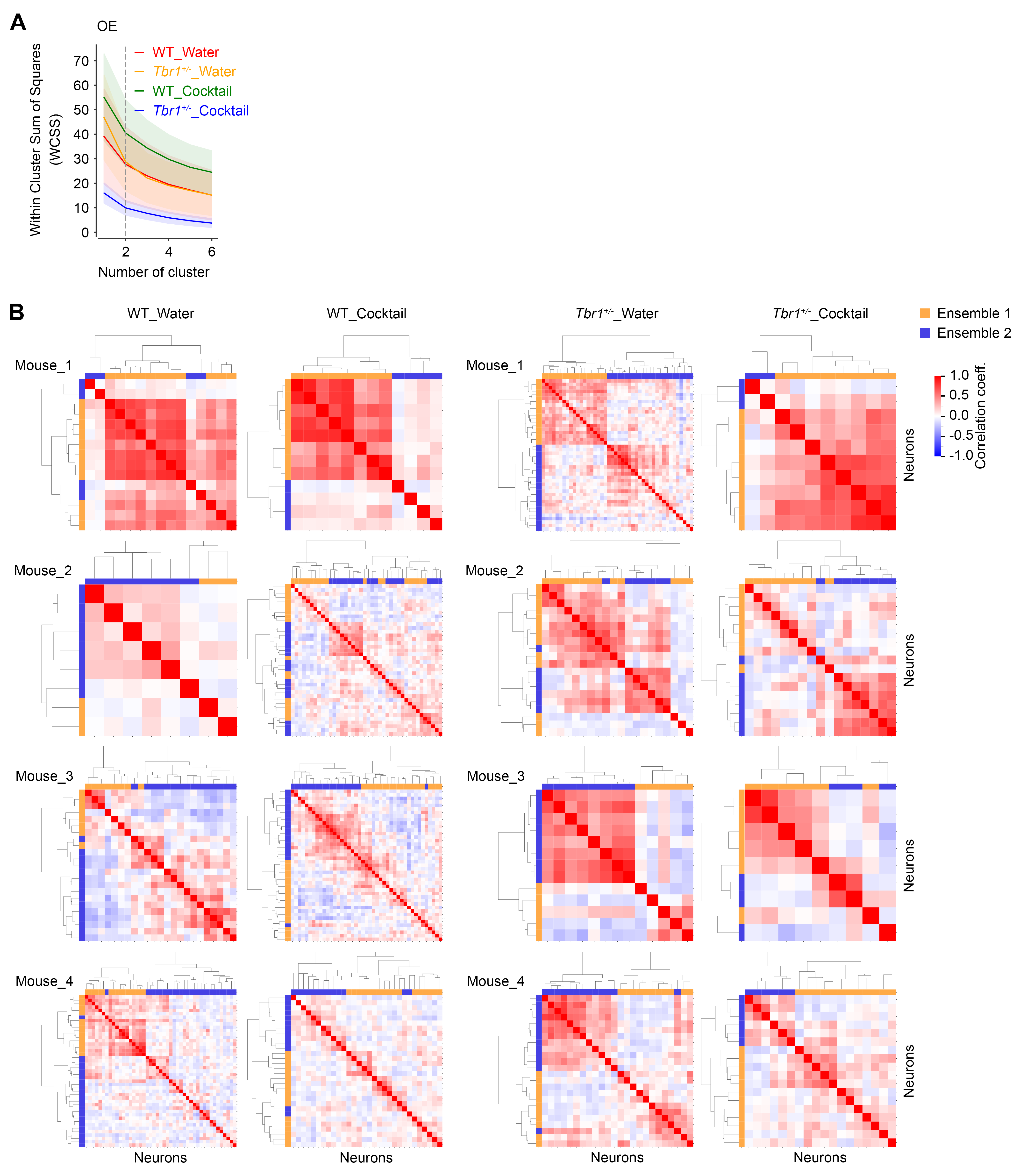

Supplement: S6 Fig — (A) The firing neurons during object exploration (OE) in the WT_Water, Tbr1+/−_Water, WT_Cocktail, and Tbr1+/−_Cocktail groups were separated into two neuronal ensembles via within-cluster sum of squares (WCSS) analysis. Data is represented as mean ± SEM. (B) Clustermap was used to represent Pearson’s correlations among neuronal activity during object exploration (OE). Related to Fig 4. (TIF) [file pbio.3003231.s006.tif]

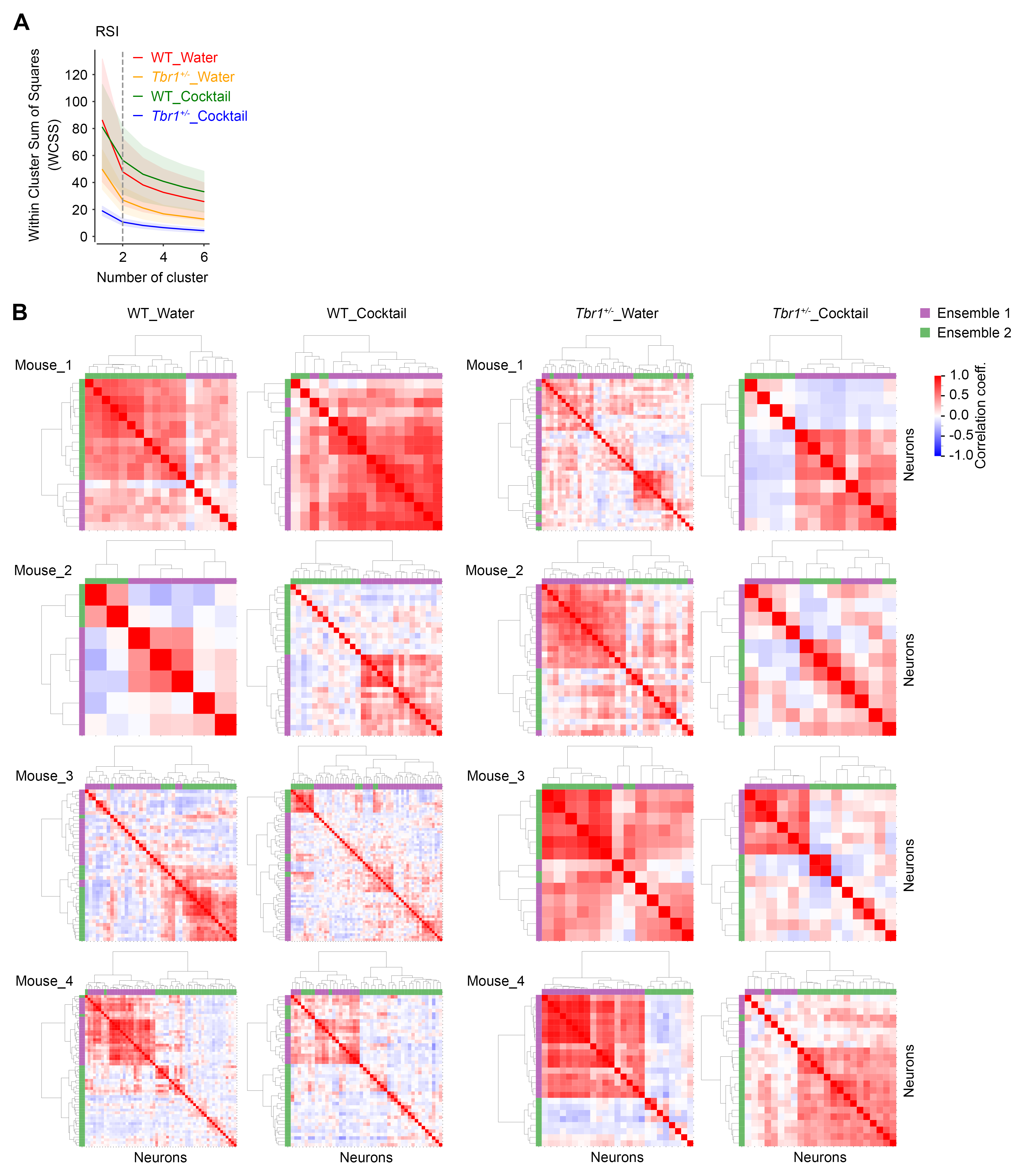

Supplement: S7 Fig — (A) The firing neurons during RSI in the WT_Water, Tbr1+/−_Water, WT_Cocktail, and Tbr1+/−_Cocktail groups were separated into two neuronal ensembles via within-cluster sum of squares (WCSS) analysis. Data is represented as mean ± SEM. (B) Cluster map was used to represent Pearson’s correlations among neuronal activity during RSI. Related to Fig 4. (TIF) [file pbio.3003231.s007.tif]

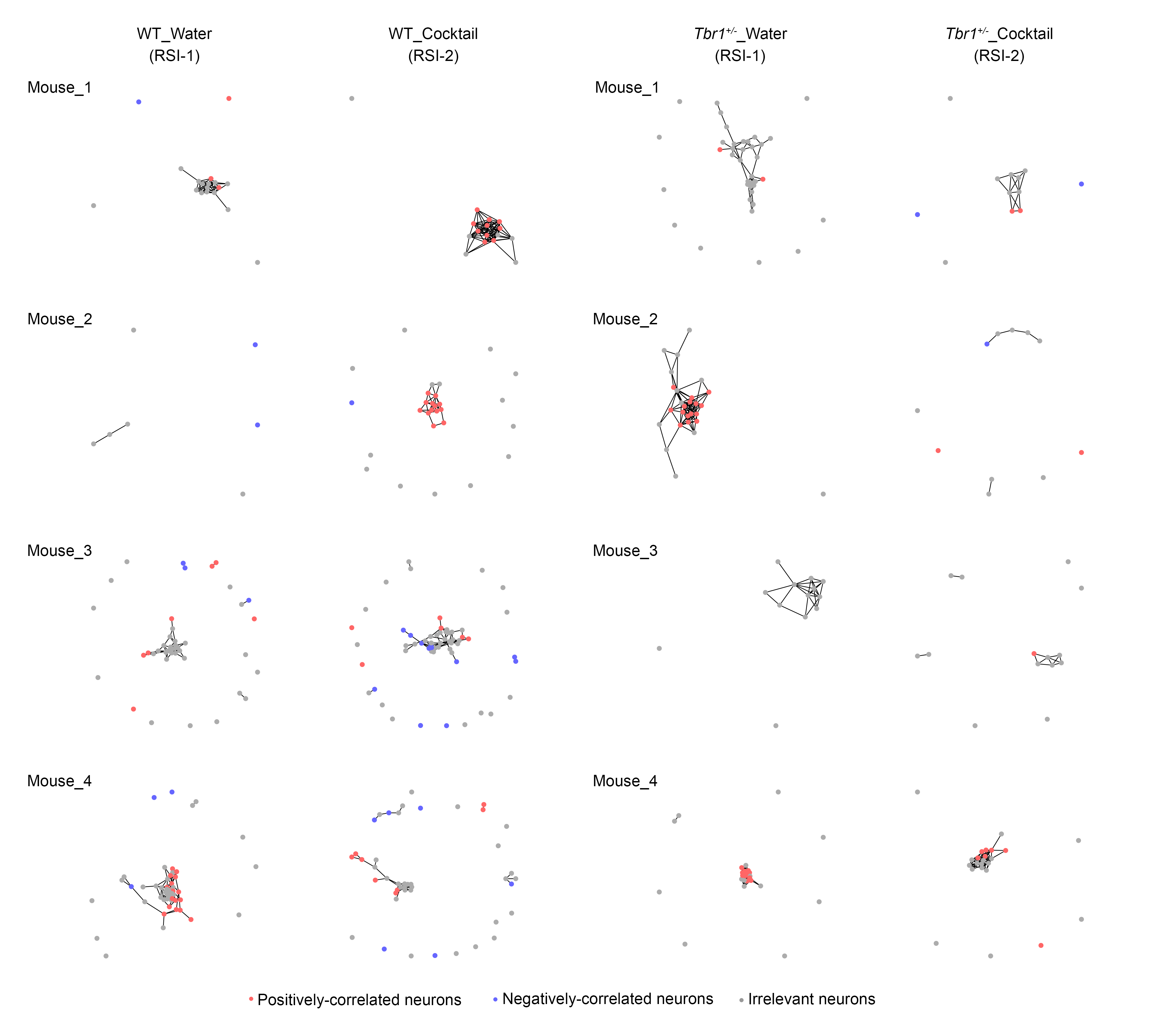

Supplement: S8 Fig — Functional networks of BLA firing neurons of individual mice (WT: n = 4; Tbr1+/−: n = 4) in RSI. Connections (lines) between the nodes indicate a significant similarity in activation patterns relative to shuffled data (10,000 permutations via phase randomization). Red, neuron positively correlated with sociality. Blue, neuron negatively correlated with sociality. Gray, neuron irrelevant to social behavior. WT mouse #3 and Tbr1+/− mouse #4 are also shown in Fig 6B. The data underlying the graphs shown in this figure can be found in the S3 Data. (TIF) [file pbio.3003231.s008.tif]

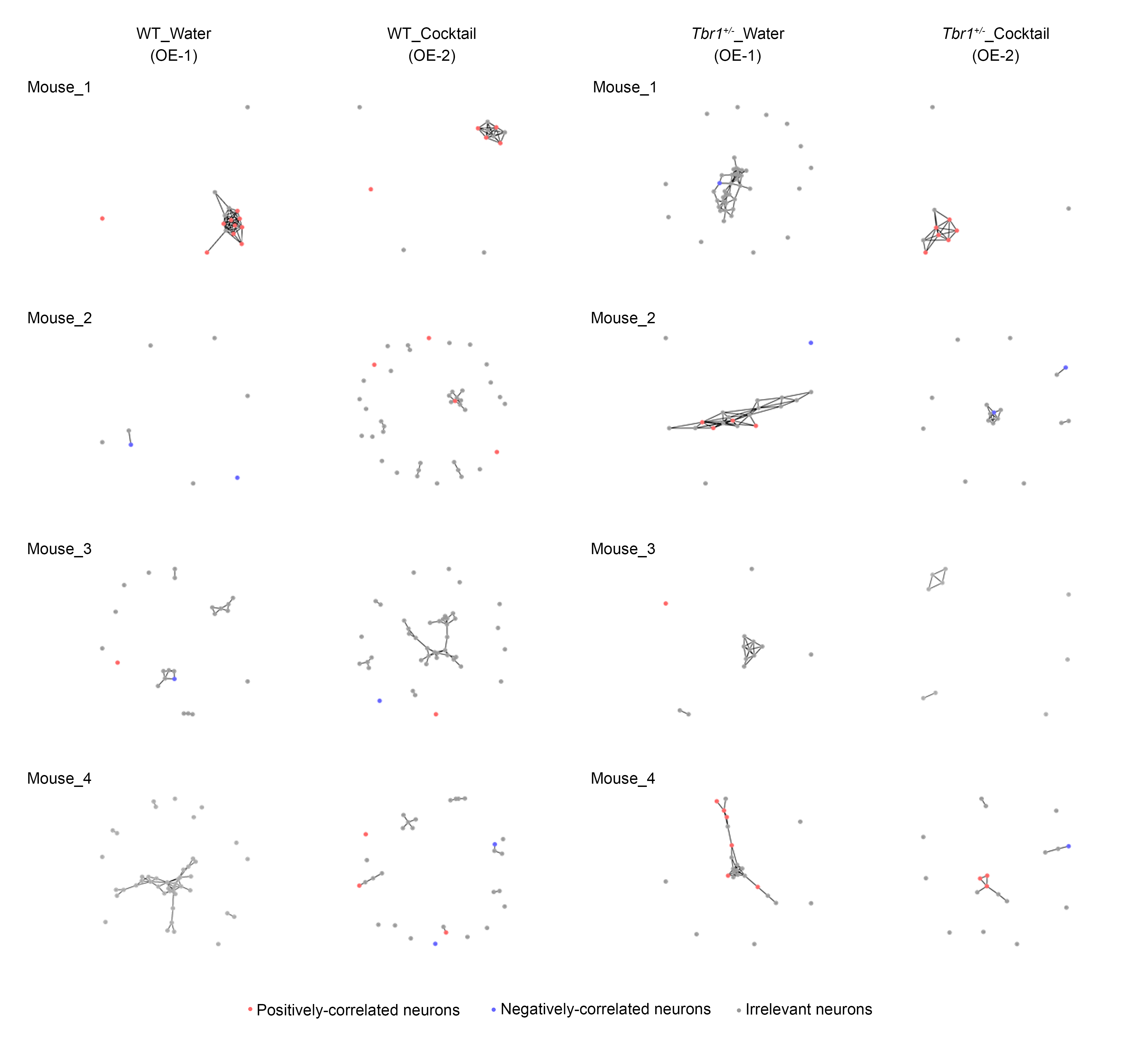

Supplement: S9 Fig — Functional networks of BLA neurons identified during the optical exploration (OE) test. Connections (lines) between nodes indicate a significant similarity in activation patterns relative to shuffled data (10,000 permutations via phase randomization). The red and blue nodes represent neurons positively or negatively associated with object exploration. The gray nodes represent neurons irrelevant to approaching behaviors. WT mouse #3 and Tbr1+/− mouse #4 are also shown in Fig 6D. The data underlying the figure can be found in the S3 Data. (TIF) [file pbio.3003231.s009.tif]

GRIA1

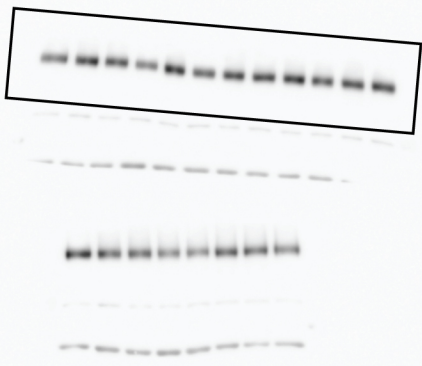

HOMER1

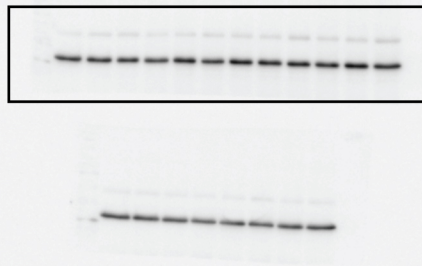

GRIN2A

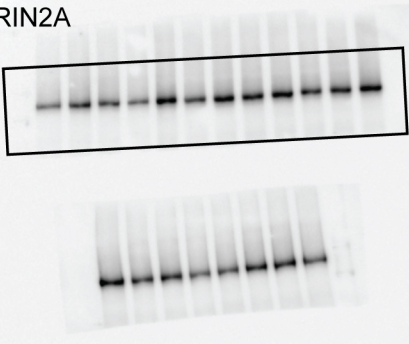

GABBR2

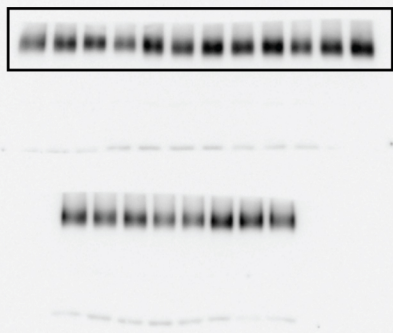

GRIN2B

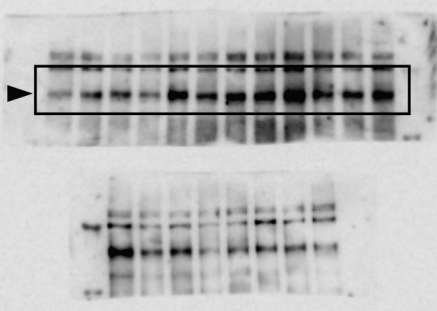

SYNPO

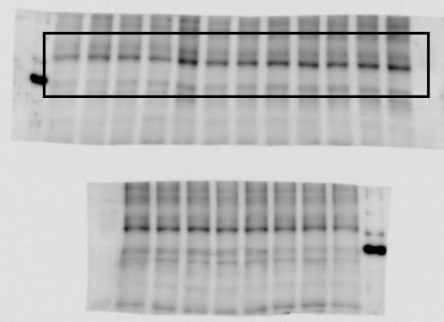

LIN7

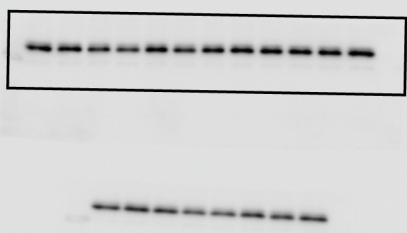

SAP97

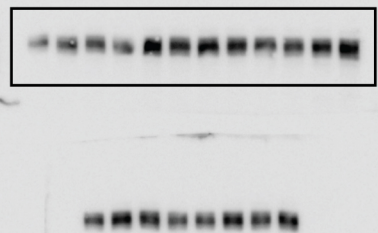

DPYSL3

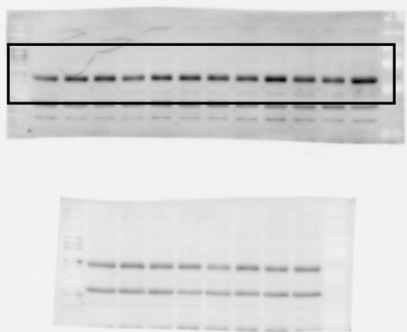

Coomassie Blue

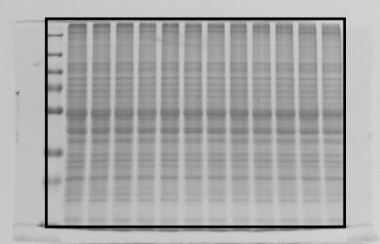

Supplement: S1 Raw Images — (PDF) [file pbio.3003231.s014.pdf]
