# Supplementary material for: Low-dose mixtures of dietary nutrients ameliorate behavioral deficits in multiple mouse models of autism
Source: PLoS Biol. 2025 Dec 2;23(12):e3003231. doi: 10.1371/journal.pbio.3003231 (PMC12671748; doi:10.1371/journal.pbio.3003231)
Supplement: Attachment — Submitted filename: Rebuttal20251008.pdf [file pbio.3003231.s017.pdf]

Our point-by-point response

Reviewer #1:
This is a very strong study that shows the role of nutrients in restoring ASD-linked deficits in the Tbr1+/- mouse model. Huang et al. provides a compelling narrative on the benefits of nutrient supplements with BCAA, L-Serine and Zinc for improving social behaviors in the Tbr1+/- mouse model. Through a combination of proteomics, calcium imaging, and behavioral imaging, Huang et al. highlight the behavioral improvements seen in Tbr1+/- mice upon administration of this combined nutrient supplement.
Major results from this set of experiments are as follows:
1. Proteomic analysis from LC-MS-MS experiments revealed a difference in the Tbr1+/- group treated only with water (as evidenced by PCA analysis in Figure 1), suggesting that cocktail administration rescues the protein composition of brain lysates (becoming more similar to the wild-type groups) in the Tbr1+/- cocktail group. Additionally, gene analysis showed that cocktail treatment affects genes involved with synapses and mitochondrial activity.
2. Calcium imaging revealed that: (1) Tbr1+/- mice have stronger correlations between individual neurons and their ensembles, regardless of the treatment or task (in both open exploration and reciprocal social interaction), (2) Tbr1+/- mice treated with the nutrient supplement had a decrease in the positively-correlated neurons and an increase in the negatively correlated neurons in the reciprocal social interaction task, (3) Tbr1+/- mice treated with the nutrient supplement mixture have decreased mean activity in reciprocal social interaction, suggesting that this mixture reduces hyperactivity, and (4) the neurons that respond to the reciprocal social interaction task change as a result of nutrient supplementation. From these results and the additional information shared in the paper, the authors have clear evidence that points towards a rescue of the hyperactivity seen in the BLA network as a result of supplementation with this mixture of nutrients in Tbr1+/- mice.
3. Lastly, using behavior experiments, the authors confirm that the mixture of nutrients at lower doses has superior effects to using individual nutrients in the reciprocal social interaction task. They demonstrate this finding in other mouse models as well, including the Cttnbp2+/M120I model. They also perform other behavioral experiments - including the three-chamber test, the auditory fear conditioning test, as well as the open field and elevated plus maze tests. Their findings are in line with the references they cite, suggesting that there is an added benefit to combining these nutrients instead of providing supplementation with individual nutrients.

Overall, this study includes controls to show that the effects seen in the Tbr1+/-mice are valid. Although the authors state that there were no calculations done to determine sample sizes, it seems as if there are sufficient sample sizes for each experiment, and the authors state that they have used similar sample sizes in previous studies.
Minor weaknesses:
1. In the introduction, an additional explanation for the selection of the two additional ASD mouse models would be helpful to understand the rationale behind selecting those specific models as positive controls.
2. The authors should revise this paper to ensure that figures are referenced within the correct sections. For example, in the paragraph 233-239, the reference to Figure 3E should be to Figure 2E. Similarly, in the following paragraph (Lines 240-250), both references to Figure 3F should be to Figure 2F. Again, in the next result section (lines 258-270), the reference to Figure 4C should be to Figure 3C.

Other than these minor comments, this paper provides a strong narrative for the benefit of combined

nutrient supplement in correcting the social behaviors seen in the Tbr1+/- ASD mouse model.

Our response to Reviewer #1's comments
We greatly appreciate Reviewer #1's strong support and positive feedback. Regarding the two minor concerns raised, we have now clarified in the Introduction (Lines 131-147) our rationale for selecting *Nf1*$^{+/-}$ and *Cttnbp2*$^{+/M120I}$ mice as additional models to evaluate the beneficial effects of our supplement cocktail. We also thank Reviewer #1 for pointing out the figure citation errors in the text. Given that we have now rearranged original **Fig. 1** to be **new Figs. 1 and 2** and added more immunoblotting data as **new Fig. 3**, all figure numbers have been changed accordingly. We have carefully corrected the figure citations in the revised manuscript. We sincerely thank Reviewer #1 for their thoughtful comments and supportive evaluation of our work.

Reviewer #2's general comments:
Based on numerous prior studies from this group and others showing the beneficial effects of single supplements (serine, BCAA, zinc) in ASD models (ref. 10, 12-18, 23-27), the current study tested the effects of a cocktail of dietary supplements using proteomics, in vivo Ca2+ imaging and behavioral assays. While the experiments are carefully designed and conducted, the conceptual novelty of this study is limited. Furthermore, there are a few concerns that need to be addressed.

Our response to Reviewer #2's general comment.
We thank Reviewer #2 for her/his comments on our manuscript. Our responses to her/his specific comments are provided below.

Reviewer #2's specific comment #1. Proteomics studies focus on one of the co-expression modules (Black), and did GO and STRING analyses (Fig. 1). However, it is unclear how these synaptic proteins are altered by Tbr1 haploinsufficiency and cocktail supplementation. Are they significantly reduced in Tbr1+/- mice and rescued by cocktail supplementation? The expressional changes in key proteins need to be confirmed.
Reviewer #2's specific comment #2. The conclusion that "the total proteomes of Tbr1+/- mouse brains are different from those of WT brains and that cocktail supplementation makes the protein expression profiles of Tbr1+/- mice more comparable to those of WT mice" is mainly based on PCA plots, which is not adequate. More in-depth analyses are needed to show the impact of cocktail supplementation on protein expression in this ASD model.

Our response to Reviewer #2's specific comments #1 and #2.
Since Reviewer #2's comments #1 and #2 are both relevant to **original Fig. 1**, we respond to them together as follows:

In the current study, we formulated supplement cocktails using lower concentrations of individual nutrients. Given that the levels of particular nutrients were significantly reduced, we expected their effects on specific targets to be limited. However, the combined influence of all supplements on functionally relevant protein networks may still be substantial. Based on this rationale, we did not attempt to identify individual proteins with altered expression levels, which is why such an analysis was not conducted in the original manuscript. Instead, we performed two analyses for our proteomic results, one is PCA, as pointed out by Reviewer #2, and the other is a weighted gene co-expression network analysis (WGCNA).

The PCA analyzes the major differences among the four samples. Although the molecular composition and characteristics are unknown, the results of the PCA indicate that the ¼ cocktail treatment for 7 days rendered the *Tbr1*$^{+/-}$ and WT proteomes more comparable to each other, and that the Tbr1_water group was relatively distinct from the other three groups. This data indicates the effect of the supplement cocktail on total proteomes.

In terms of the WGCNA, it analyzes the correlation among all proteins in a system rather than the differences of individual proteins in isolation. We feel it is a perfect analytical approach for our experiment because we did not expect to observe huge changes in individual proteins arising from our low-dose supplement cocktail treatment. WGCNA identifies clusters (or modules) by grouping proteins displaying correlations, even if the changes in particular proteins are small. Our results revealed that the "Black" module, comprising 271 proteins, exhibits the highest correlation and statistical significance ($P = 0.0$) with the treatment, which is consistent with our PCA analysis showing that supplement cocktail-treated groups were clearly separated from water groups.

We apologize for not including detailed information about the Black module in the original manuscript. To further elucidate the changes in the Black module, we have rearranged the figures related to our bioinformatic analysis. To emphasize the rationale for combining zinc, BCAA, and serine into a supplement cocktail, we have converted **original Fig. 1A** into **new Fig. 1**. The PCA analysis in **original Fig. 1B** has become **new Fig. 2A**. **New Fig. 2B and 2C** contain the WGCNA results (module-trait relationship heatmap and eigengene expression levels). **New Fig. 2D** is the new network analysis of all Black module proteins using STRING. Below, please see **new Figure 2** as it appears in the revised manuscript for Reviewer #2's information.

The results show that the Black module was upregulated by the supplement cocktail treatment. Expression levels of the proteins in the Black module were reduced in the water-treated groups but increased in supplement-treated groups, regardless of genotype, although *Tbr1*$^{+/-}$ mice seem to present a more promising impact. The entire protein list and the connectivity and relative protein levels of all 271 proteins are summarized in **new S2_Data**.

Based on GO analysis using PyWGCNA and STRING, synapse-related GO—including synapse organization, modulation of chemical synaptic transmission, synaptic signaling, and regulation of synapse organization—were the top GO terms associated with the Black module (**new Fig. 2D and S2_Fig**). Since we use total lysates for proteomic analysis, the enrichment for synapse-related proteins in the top module in the WGCNA provides evidence for the impact of the supplement cocktail on synaptic protein expression. In addition to synapse-related proteins, three other GO terms, i.e., generation of precursor metabolites and energy, vesicle-mediated transport, and cytoskeleton organization, were significantly associated with the Black module (**new Fig. 2D**). Proteins involved in both excitatory and inhibitory neurotransmission were present in the Black module (**new S4_Fig**). Moreover, the 271 proteins in the Black module were highly connected to each other based on the STRING functional network (**new Fig. 2D**). Thus, supplement cocktail treatment may upregulate expression of these proteins to modulate synaptic activity and function and even network-level connectivity.

Note that we did not find the modules to be significantly associated with genotype (correlation <-0.8 or >0.8 and P < 0.05). Perhaps TBR1 primarily functions during development, influencing neuronal migration and axonal projection of the cerebral cortex and basolateral amygdala, ultimately altering neuronal connectivity. In adults, TBR1 protein levels are reduced to a very low level, and its impact on gene expression becomes limited. Thus, although neuronal connections have been changed, global gene expression may not be noticeably altered in adult *Tbr1*$^{+/-}$ mice.

New Fig. 2 (see the revised manuscript for details)

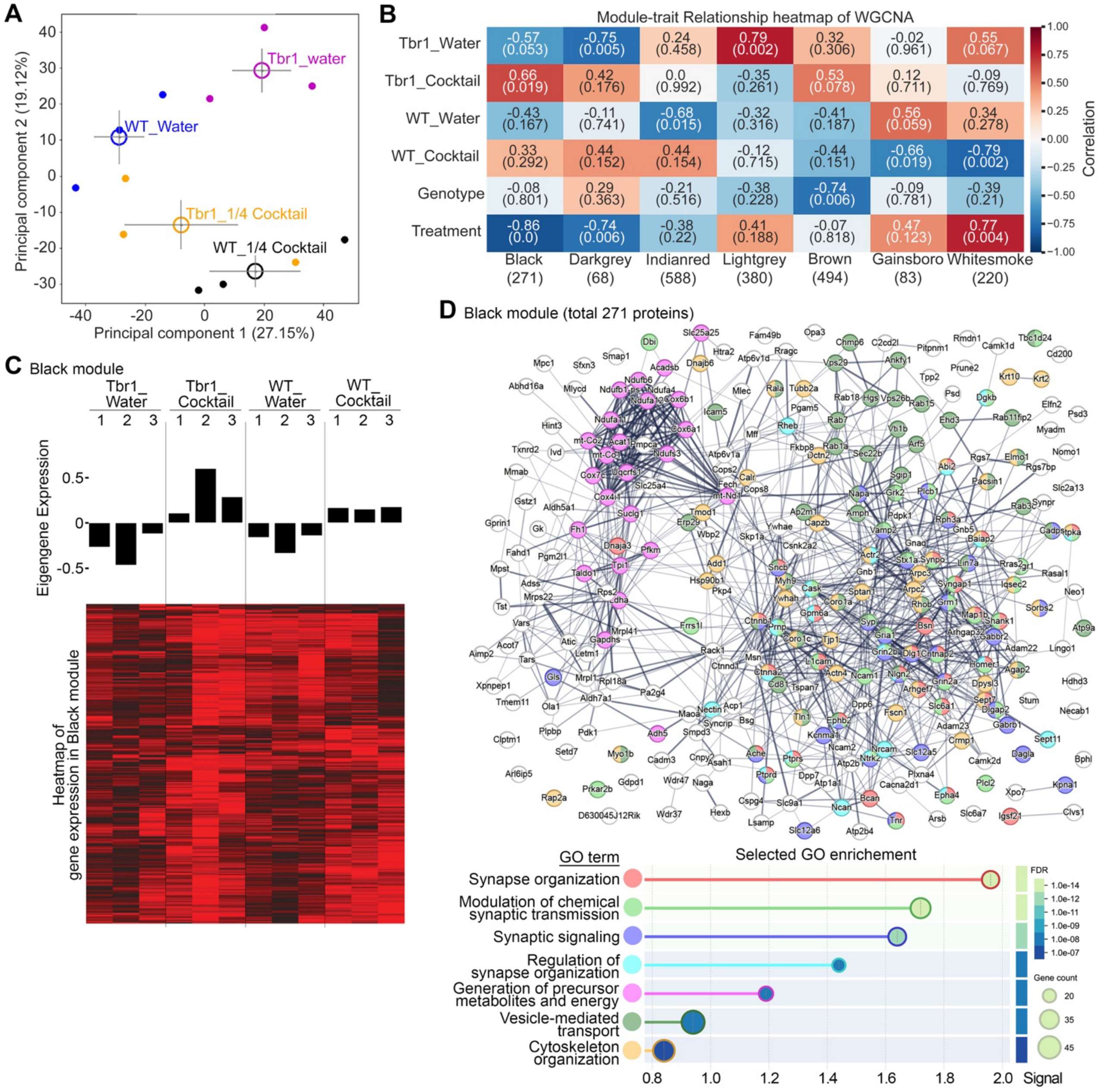

Since we expected that changes in individual target proteins would be small and considering the relatively low sensitivity of immunoblotting, we originally did not perform immunoblotting as validation of LC-MS-MS. In response to Reviewer #2’s suggestions, we have picked several

proteins from the Black module list (**new S2_Data**) based on their functions and fold-change values, as well as antibody availability, for immunoblotting validation. We examined by immunoblotting a total of **9** proteins in the Black module, including GRIA1, GRIN2B, GRIN2A, LIN7, DPYSL3, Homer1, GABBR2, SAP97, and SYNPO (**new Fig. 3)**. Two-way ANOVA and post-hoc testing were performed to analyze the cocktail treatment effect and response of *Tbr1*$^{+/–}$ and WT mice to cocktail treatment. Among all of the examined proteins, the levels of five (i.e., GRIA1, GRIN2B, GRIN2A, DPYSL3, Homer1) were significantly increased after supplement cocktail treatment for 7 days, although the increments were only in the order of 10-30%. In addition, GRIA1, GRIN2b, DPYSL3 and Homer1 had higher protein levels in the Tbr1+/–_cocktail group compared to the Tbr1+/–_water group (**new Fig. 3**). Given that these proteins are critical for synaptic activity and plasticity, upregulation of these proteins upon supplement cocktail treatment suggests that the treatment may improve synaptic activity and plasticity of *Tbr1*$^{+/–}$ mice via these proteins.

The above explanations and modifications have now been integrated into the revised manuscript on **PP. 6-8**. The changed parts are highlighted in blue font.

Reviewer #2's specific comment #3. Fig. 2, what is the relevance of the "stronger correlation between individual BLA neuron activity and its corresponding ensemble" in Tbr1+/- mice to their behavioral deficits?

Our response to Reviewer #2's specific comment #3.
A stronger correlation indicates that the individual neuron is a crucial component of the ensemble's function, such as in social interaction or object recognition, in our experiments. Those neurons more consistently participate in the coordinated activity of their group. Although it is an interesting observation about *Tbr1*$^{+/–}$ neurons, this property may not be relevant to behavioral deficits in *Tbr1*$^{+/–}$ mice per se because it is not specific to social behavior. We detected a stronger correlation in both OE and RSI of *Tbr1*$^{+/–}$ mice when compared to WT littermates (**new Fig. 4D**). Moreover, nutrient supplementation did not alter the stronger correlation between individual neuron activity and its corresponding ensemble in *Tbr1*$^{+/–}$ mice (**new Fig. 4D**). To investigate which deficits in calcium imaging are relevant to behavioral deficits and nutrient supplementation, we performed additional analyses and found that other properties were more relevant to behavioral deficits and nutrient supplementation, the results of which are shown in **new Figs. 4E-4F, 5, and 6**. First, we analyzed the activity correlation of repetitively responsive neurons (**new Fig. 4E-4F**). Second, we assessed the change in activity of BLA neurons (**new Fig. 5**). Finally, we determined the functional neural network of the BLA (**new Fig. 6**). To make our point clearer, we have integrated the above explanation into **PP. 8-9** of the revised manuscript.

Reviewer #2's specific comment #4. Fig. 3C, it is unclear whether the differential neuronal responses to social stimulation by Tbr1 haploinsufficiency or cocktail supplementation are statistically and biologically significant. How could Tbr1 haploinsufficiency alter the cell type responding to social stimulation (line 458-459)?

Our response to Reviewer #2's specific comment #4.
For in vivo calcium imaging, we unbiasedly collected information from the individual neurons of multiple mice and used various methods to analyze the properties of those neurons. **Original Fig. 3C** (**new Fig. 5C** in the revised manuscript) summarizes the cell numbers and percentages of all

neurons whose activities are positively or negatively correlated with behaviors. Statistical analysis is not applicable to this type of quantification. Nevertheless, based on the data shown in **new Fig. 5C**, we further analyzed the mean activities of positively correlated neurons during behaviors, which are shown in **new Fig. 5D**. The results shown in **new Fig. 5D** were subjected to statistical analysis, which indicates that the activity of social-related neurons was higher in *Tbr1*$^{+/-}$ mice and that it could be corrected by cocktail treatment. These results indicate that BLA neuronal activity is altered by *Tbr1* haploinsufficiency and nutrient supplementation.

The entire paragraph containing original lines 458-459 was as follows:
"Consistent with a previous study [45], we found that very few neurons were repetitively responsive to the first and second social stimulations. Given that no negatively correlated neuron was present in RSI-1, *Tbr1* haploinsufficiency alters the cell type responding to social stimulation. This change in cell type likely contributes to the altered neural connectivity and synchronization we observed in *Tbr1*$^{+/-}$ mouse brains. However, it remains elusive how *Tbr1* haploinsufficiency changes the responsiveness of different cell types in BLA. Our observation that the supplement cocktail reduced the number of positively correlated neurons but increased the negatively correlated neurons in *Tbr1*$^{+/-}$ mice is also interesting. Accordingly, the absolute number of responsive neurons in the BLA may not be the most critical factor in controlling social behaviors. Instead, appropriate connectivity in the BLA is likely more important."

In this paragraph, we point out that "it remains elusive how *Tbr1* haploinsufficiency changes the responsiveness of different cell types in the BLA". To make this point clearer, we have revised this paragraph as follows:
"Consistent with a previous study [45], we found that very few neurons were repetitively responsive to the first and second social stimulations. Given that no negatively correlated neuron was present in RSI-1, *Tbr1* haploinsufficiency alters the cell type responding to social stimulation. This change in cell type likely contributes to the altered neural connectivity and synchronization we observed in *Tbr1*$^{+/-}$ mouse brains. However, it remains elusive how *Tbr1* haploinsufficiency changes the responsiveness of different cell types in the BLA. Dissecting the cell types by means of snRNA-seq would likely reveal the impact of *Tbr1* haploinsufficiency on BLA cell types. Our observation that the supplement cocktail reduced the number of positively correlated neurons but increased the negatively correlated neurons in *Tbr1*$^{+/-}$ mice is also interesting. Accordingly, the absolute number of responsive neurons in the BLA may not be the most critical factor in controlling social behaviors. Instead, appropriate connectivity in the BLA is likely more important. How *Tbr1* haploinsufficiency and nutrient supplementation alter cell types and modulate the neuronal response, ultimately influencing connectivity, is an intriguing topic that warrants further exploration."

Reviewer #2's specific comment #5. Fig. 3E and 3F, the extremely low number of responsive BLA neurons shared by RSI-1 and RSI-2 in WT (7 neurons) and Tbr1+/- (1 neuron) raise concerns on the data validity and interpretations.

Our response to Reviewer #2's specific comment #5.
In original Fig. 3E and 3F (**new Fig. 5E, 5F**), we attempted to investigate if the responsive neurons are used repetitively. Our observation of a low number of responsive neurons shared by the two RSIs indicates that the majority of BLA neurons are not repetitively activated nor involved in the same type of behaviors. This observation is consistent with a previous study conducted by Dr.

Andreas Lüthi's laboratory (Fustiñana et al., 2021, Nature 592:267). This point had been mentioned and the respective reference was cited in the original manuscript. For Reviewer #2's information, we highlight it in the revised manuscript with blue font (**P. 11**).

Reviewer #2's specific comment #6. How to link Tbr1 deficiency-induced "hyperactivity and hyperconnectivity of BLA neurons during social interaction" to the social deficits in Tbr1+/- mice? The degree of whole-brain synchronization (connectivity) of the BLA is reduced by Tbr1 haploinsufficiency [43]. What is the effect of dietary cocktail on the correlated neuronal activity between BLA and other brain regions?

Our response to Reviewer #2's specific comment #6.
Both hyper- and hypo-connectivity is associated with ASD. They both disrupt normal information flow in the brain upon specific stimulation and they result in inappropriate responses to stimulation. The BLA is known to be a critical brain region for social and emotional behaviors. It is also highly sensitive to *Tbr1* haploinsufficiency (Huang et al., 2014, Nature Neuroscience 17:240). We have shown previously that although numbers of C-FOS-positive cells are generally higher in *Tbr1*$^{+/-}$ mice, whole-brain synchronization of the BLA is reduced in *Tbr1*$^{+/-}$ mice, indicating impaired functional connectivity of the BLA with other brain regions. However, it is unclear if the intra-BLA connectivity related to social interaction is also impaired by *Tbr1* haploinsufficiency. In the current study, we endeavored to use in vivo calcium imaging to investigate the neuronal response of the BLA during social behaviors and analyzed if *Tbr1* haploinsufficiency influences intra-BLA connectivity during social behaviors. Our data clearly show that *Tbr1* haploinsufficiency results in hyperactivity of BLA neurons and hyperconnectivity among BLA neurons during social behavior. Moreover, these kinds of abnormal connectivity were corrected by supplement cocktail treatment. Note that the impaired social behaviors of *Tbr1*$^{+/-}$ mice were also improved by supplement cocktail treatment. Thus, the hyperactivity and hyperconnectivity of BLA neurons during social interaction is relevant to the social deficits displayed by *Tbr1*$^{+/-}$ mice. Together with our previous results of reduced whole-brain connectivity of the BLA in *Tbr1*$^{+/-}$ mice (Hsu et al., 2024, PLOS Biology 22:e3002646), our data indicate that both intra-BLA and whole-brain BLA connectivity are affected by *Tbr1* haploinsufficiency, which are critical for social behaviors.

Certainly it would be interesting to investigate the effect of the supplement cocktail on whole-brain connectivity of the BLA in *Tbr1*$^{+/-}$ mice. However, we feel it is beyond the scope of the current study. In the original manuscript, we did mention that "<u>It would be intriguing to investigate in the future if whole-brain connectivity can also be corrected by dietary supplementation</u>", which has been highlighted in blue font in the revised manuscript for Reviewer #2's information (**P. 16**).

Reviewer #2's specific comment #7. Proteomic and in vivo Ca2+ imaging tests were only done in one ASD model (Tbr1+/- mice). The title and abstract gave the impression that dietary cocktail ameliorated the altered proteins and neural connectivity across multiple ASD models, which needs to be corrected. It is simply unknown whether other ASD models have abnormal synaptic proteomes and impaired neural ensembles in BLA that are similar to Tbr1+/- mice, and whether the dietary mixtures have any effects on proteomes and neural ensembles in these models.

Our response to Reviewer #2's specific comment #7.

We have modified the title of the revised manuscript to “Dietary nutrient mixtures ameliorate synaptic proteome abnormalities, neural ensemble dysfunction, and behavioral deficits in the *Tbr1* mouse model of autism”.

Reviewer #2’s specific comment #8. The authors demonstrated previously that 0.45% BCAA or 40 ppm zinc supplemented is sufficient to improve the social behaviors of Cttnbp2+/M120I mice [13, 14, 18], while this study showed the lack of effects with .225% BCAA or 20 ppm zinc (Fig. 5D). What makes such a big difference by just reducing the dose by half?

Our response to Reviewer #2’s specific comment #8.
Perhaps the thresholds of the beneficial effects of individual BCAA and zinc treatments are between 0.225-0.45% and 20-40 ppm for *Cttnbp2*$^{+/M120I}$ mice, respectively, under our experimental conditions. Thus, 0.45% BCAA or 40 ppm zinc elicits a beneficial effect, unlike the treatment with 0.225% BCAA or 20 ppm zinc.

In our study, to determine the appropriate concentrations of individual nutrients, we applied the concept of classical two-fold series dilution for biochemical analysis and designed 1/2, 1/4 and 1/8 cocktails. If individual supplements do not have a beneficial effect on social behaviors, but a mixture does, then mixing the nutrients likely triggers a synergistic effect.

Reviewer #2’s specific comment #9. Fig. 6E, is there any significant difference between Tbr1+/- with water and Tbr1+/- with ¼ cocktail? If not, the conclusion "Thus, the 1/4 cocktail treatment rescued the memory performance of Tbr1+/- mice" is overrated.

Our response to Reviewer #2’s specific comment #9.
We thank Reviewer #2 for raising this point. Since the memory performance of *Tbr1*$^{+/–}$ mice was not different from that of WT littermates, the description has been revised as follows:
“Thus, the 1/4 cocktail treatment improves the memory performance of *Tbr1*$^{+/–}$ mice to a level comparable to that of WT littermates.”

Reviewer #2’s specific comment #10. Why were 1/4 cocktail, 1/8 cocktail or 1/2 cocktail used in behavioral assays (Fig. 5, & 6)? Do different ASD models need different doses of the supplement mixture? If so, what is the potential reason?

Our response to Reviewer #2’s specific comment #10.
To demonstrate the synergistic effects of nutrient cocktails, we conducted a two-fold dilution to determine the minimal concentrations of individual nutrients in the mixtures. In our analyses, we found that the 1/2 cocktail improved the social interaction of all examined ASD models. For *Tbr1*$^{+/–}$ mice, the 1/4 cocktail was still effective. The lowest dose, i.e., the 1/8 cocktail, proved effective in *Cttnbp2*$^{+/M120I}$ mice. Thus, different minimal doses of the supplement mixture were effective in different ASD models.

We speculate that the different doses may be relevant to the function and/or phenotypic severity of ASD mutations. *Tbr1* haploinsufficiency disrupts axonal projection of the BLA and results in a reduction or absence of the anterior commissure in both humans and rodents. These neuroanatomical deficits occur in all individuals possessing *TBR1* mutation (Huang et al., Nature

Neuroscience 17:240; Nambot et al., 2020, European Journal of Human Genetics 28:770). Similar to *Tbr1*$^{+/-}$ mice, *Cttnbp2*$^{+/M120I}$ mice also exhibit typical ASD-linked behavioral deficits. However, unlike *Tbr1*$^{+/-}$ mice, *Cttnbp2*$^{+/M120I}$ mice do not present recognizable neuroanatomical deficits. When an ASD mutation causes structural defects, it would require a higher dose of supplement cocktail to rescue the behavioral deficits. This speculation has now been included in the Discussion section (**P. 17**) of the revised manuscript. We thank Reviewer #2 for this point.

Reviewer #2's specific comment #11. Given what has been extensively reported on single supplements, the current study does not provide new insights into the significant benefits of dietary mixtures. What are the side effects of single supplements? What makes dietary mixtures more beneficial? What is the reason underlying the better effects of dietary mixtures than single supplements?

Our response to Reviewer #2's specific comment #11.
There are potentially significant side-effects of high-dose zinc and BCAA supplements, as follows:

(1) Zinc (Mayo Clinic, https://www.mayoclinic.org/drugs-supplements-zinc/art-20366112, the following information is derived from the Mayo Clinic's website)
High-dose zinc supplementation taken by mouth may cause diarrhea, dizziness, headache, nausea, upset stomach, and vomiting. Long-term high-dose zinc supplementation can also lead to copper deficiency because zinc and copper share the same transporter. People with low copper levels might have brain and nervous system issues, as well as numbness and weakness in the arms and legs.

(2) BCAA and Serine (Fernstorm, 2005, J. Nutr. 135:1546S; Holeček, 2022, Physiol. Res. 71:29; https://www.urmc.rochester.edu/encyclopedia/content?contenttypeid=19&contentid=Serine)
In general, single amino acid supplementation is not recommended because of negative nitrogen balance. It may reduce metabolism efficiency and result in kidney issues. Due to an imbalance of amino acids, single amino acid supplementation may also cause growth problems in children. Moreover, the uptake and transportation of amino acids require specific transporters located on the plasma membrane. Branched-chain amino acids and other aromatic amino acids (including tryptophan, tyrosine, and phenylalanine) share the same transporter. Thus, these two subtypes of amino acids compete with each other for transportation. Long-term high-dose supplementation of branched-chain amino acids may influence brain function by reducing aromatic amino acid transport at the blood–brain barrier. Since aromatic amino acids are the precursors for critical neurotransmitters, such as serotonin and catecholamines, reducing the concentration of aromatic amino acids has functional consequences in terms of altering hormonal function, blood pressure, and affective state.

Considering these potential side-effects of long-term high-dose zinc and amino acid supplementation, we proposed **low-dose dietary supplement mixtures** for long-term treatment of ASD conditions. In the Introduction of our original manuscript, we pointed out that there are two advantages in using this strategy. First, reducing the potential side-effects of long-term high-dose treatment with a single nutrient. Based on previous studies, dietary therapy has to be conducted continuously to maintain the beneficial effects. For ASD treatment, it is better to perform the intervention as early as possible. Therefore, the timescale for dietary therapy of ASD is the entire lifespan. It is certainly prudent to minimize all potential side-effects caused by high-dose

supplementation of a single nutrient. To make this point clearer, we have now included the aforementioned side-effects of high-dose single-nutrient supplementation in the **Introduction** and **Discussion** of the revised manuscript.

The second advantage is to broaden the targets of the combined supplements. Since synaptic alteration is one of the features of ASD, the improvements in synaptic function and activity may be generally beneficial for ASD patients. Given that zinc, BCAA, and serine may improve synaptic function and activity via different mechanisms, a supplement mixture containing zinc, BCAA, and serine may ameliorate to varying extents the ASD-linked deficits. Thus, the treatment may not be limited to a specific mutation. Even if only partial improvement is realized, it would still be beneficial to patients and their families. This second point is also now emphasized in the Discussion section of the revised manuscript.

Reviewer #2's specific comment #12. There are a few errors when referring to Figures (e.g. should be Fig. 2E (line 238), Fig. 2F (line 246), Fig. 2F (line 248), Fig. 3C (line 268)).

Our response to Reviewer #2's specific comment #12.
Apologies. We have corrected the figure citation errors in the text. We thank Reviewer #2 for her/his comments and suggestions.

Reviewer #3:
This is an interesting and well-executed manuscript demonstrating that Tbr1 haploinsufficiency leads to hyperactivation and increased neuronal connectivity within the basolateral amygdala (BLA). To counteract these abnormalities, the authors developed nutritional supplement cocktails containing zinc, branched-chain amino acids (BCAAs), and serine—nutrients known to influence synapse formation and neuronal function—which were administered to Tbr1+/- mice. This intervention altered both the synaptic and metabolic proteomes and restored BLA activity and functional connectivity during social interactions. Importantly, while low doses of individual nutrients had no significant effect on social behaviour, their combination in supplement form significantly improved social behaviour and associative memory in Tbr1+/- mice, suggesting a synergistic effect. Moreover, the supplement cocktails also ameliorated social deficits in two additional ASD mouse models, Nf1+/- and Cttnbp2+/M120I, indicating potential for broader therapeutic application.
While the experimental design is generally sound, a critical issue should be addressed before the manuscript is suitable for publication. Specifically, although the authors compellingly demonstrate BLA hyperactivity in the Tbr1+/- model and show that the supplement cocktail modulates both neuronal activity and the synaptic proteome, the mechanistic link between these proteomic changes and the observed restoration of BLA connectivity remains unclear. Providing further clarification or additional data to establish a causal relationship between the proteomic alterations induced by the supplement and the normalisation of network-level connectivity would significantly strengthen the manuscript.

Our response to Reviewer #3:
We very much appreciate the positive comments and strong support of Reviewer #3. We have significantly revised the proteomic data in the revised manuscript by presenting more analyses in **new Figs. 2 and 3** and **S4_Fig**.

In brief, we have moved the module-trait relationship heatmap of WGCNA from **original Supplementary Fig. 1** to **new Fig. 2B** to highlight our use of WGCNA in this study. We then show the eigengene expression of the Black module of our WGCNA in **new Fig. 2C and S2_Data**. The results clearly show that supplement cocktail treatment enhances the expression of Black module eigengenes in both *Tbr1*$^{+/-}$ and WT littermates, although the increment seems more marked in *Tbr1*$^{+/-}$ mice. To understand the function of Black module eigengenes, we further subjected all 271 proteins listed in the Black module to GO and functional network analyses, as shown in **new Fig. 2D** and **S1-S4_Figs**. Based on the STRING functional protein network, we found synapse-related GO—including synapse organization, modulation of chemical synaptic transmission, synaptic signaling, and regulation of synapse organization—were the top GO terms associated with the Black module (**new Fig. 2D**). Note that we used total lysates for our proteomic analysis. The enrichment of synapse-related proteins in the top module in the WGCNA supports the impact of the supplement cocktail on synaptic protein expression. In addition to synapse-related proteins, three other GO terms, i.e., generation of precursor metabolites and energy, vesicle-mediated transport, and cytoskeleton organization, were significantly associated with the Black module (**new Fig. 2D**). Proteins involved in both excitatory and inhibitory neurotransmission were also present in the Black module (**new S4_Fig**). Moreover, these 271 proteins in the Black module were highly connected to each other based on the STRING functional network (**new Fig. 2D**). Thus, our supplement cocktail treatment may upregulate the expression levels of these proteins to modulate synaptic connections and even network-level connectivity. Using immunoblotting, we have further confirmed upregulation of GRIA1, GRIN2A, GRIN2B, Homer 1, and DPYSL3 upon supplement cocktail treatment. Given that these proteins are critical for synaptic activity and plasticity, our results are consistent with the observed improvement in neural ensembles and social behaviors in mice. These new data have been included in the revised manuscript as **new Fig. 3**. Further description of immunoblot validation is included on **PP. 7-8** of the revised manuscript.
